# Physiological and Transcriptional Responses of the Blood Clam *Anadara kagoshimensis* (Tokunaga, 1906) During Cadmium Exposure

**DOI:** 10.3390/toxics14100883

**Published:** 2026-09-30

**Authors:** Yuxin Ren, Fei Li, Xindong Xing, Haocheng Li, Hong Yu, Huiru Liu, Zhixiong Zhou, Xiaoyu Wang, Jian Liang, Chunxiu Chen, Yongjun Guo, Shuang Liang

**Affiliations:** 1 Tianjin Key Laboratory of Aquatic Ecology and Aquaculture, College of Fisheries, Tianjin Agricultural University, Tianjin 300384, China; 2 Key Laboratory of Smart Breeding (Co-Construction by Ministry and Province, Ministry of Agriculture and Rural Affairs), Tianjin Agricultural University, Tianjin 300384, China; 3 College of Fisheries and Economy, Tangshan Maritime Institute, Tangshan 063200, China; 4 Agricultural and Rural Development Service Center of Binhai New Area, Tianjin 300452, China; 5 Tianjin Fisheries Research Institute, Tianjin 300221, China

**Keywords:** blood clam, cadmium, tissue burden, oxidative stress, RNA sequencing, biological pooling

## Abstract

Cadmium (Cd) exposure can affect metal handling, metabolism and tissue condition in bivalves on different timescales. We examined these responses in the blood clam *Anadara kagoshimensis* (Tokunaga, 1906) exposed to a nominal Cd concentration of 100 μg/L. Respiration, tissue Cd, redox biomarkers and histology were assessed over 480 h, alongside hepatopancreas RNA profiles at 0 h, 24 h and 21 d. Oxygen consumption reached its lowest exposed mean at 96 h. At 480 h, Cd concentrations were 44.23 ± 8.99 μg/g dry weight in the hepatopancreas and 31.75 ± 1.81 μg/g in the gill. SOD and CAT activities followed tissue-specific trajectories, GSH content declined, and MDA showed non-monotonic changes. Histological observations included epithelial detachment and disruption of digestive tubule structure. Descriptive expression comparisons identified metal handling, redox, cell-death and immune-related candidates, with more twofold expression differences at 21 d than at 24 h. qRT-PCR examined ten selected targets using three tank-derived RNA samples per time. Together, the physiological measurements, tissue observations and exploratory expression profiles describe a tissue- and time-dependent response to Cd exposure and identify candidates for further functional study.

## 1. Introduction

Cadmium (Cd) enters aquatic environments through industrial discharges, atmospheric deposition and terrestrial runoff, exposing resident organisms to a persistent metal contaminant [1,2,3]. In coastal aquaculture, its accumulation in shellfish is relevant both to animal condition and to the movement of contaminants through food webs. Biological effects depend on the amount of metal reaching sensitive tissues, the duration of exposure and the physiological processes that govern uptake, retention and elimination. Consequently, tissue burdens and functional responses provide complementary information about an exposure. Following both through time can reveal relationships that are obscured when toxicity is assessed from a single terminal measurement.

Bivalves are widely used to investigate biological responses to metal contamination because their tissues connect environmental exposure with several distinct physiological functions [4]. The gill has a large surface area for water exchange, whereas the hepatopancreas, also termed the digestive gland, contributes to digestion, nutrient processing and internal metal handling. Studies in *Anodonta woodiana*, *Crassostrea angulata* and *Meretrix meretrix* have documented tissue-dependent Cd accumulation and biochemical responses [5,6,7]. These differences make the gill and hepatopancreas useful complementary targets. Comparing their trajectories can help determine whether increasing internal burdens coincide with similar physiological adjustments or with contrasting patterns of antioxidant activity and tissue condition.

Redox measurements, histology and transcriptional profiles examine different aspects of this tissue response. Superoxide dismutase (SOD) and catalase (CAT) activities reflect components of enzymatic antioxidant defense, while reduced glutathione (GSH) content and malondialdehyde (MDA) provide information on antioxidant availability and lipid-peroxidation-associated changes. These endpoints can vary independently during Cd exposure [6,7,8,9]. Histological examination adds the spatial context of epithelial and digestive tubule organization. Transcript measurements further identify candidates involved in metal binding, transport, metabolism and cellular defense. Together, these measurements connect molecular candidates with the biochemical and structural characteristics of exposed tissues.

The blood clam *Anadara kagoshimensis* (Tokunaga, 1906), historically reported as *Scapharca subcrenata* (Lischke, 1869), provides an opportunity to examine these relationships in a commercially relevant bivalve [10]. Research in other clams has linked Cd exposure with metal homeostasis, metabolism and immune responses [8,11,12]. In the blood clam, salinity stress has also been associated with antioxidant and metabolic changes [13], and a chromosome-level genome is available under the name *Scapharca kagoshimensis* [14]. Despite this broader physiological and genomic context, the temporal correspondence among Cd distribution, respiration, redox measurements and tissue architecture remains incompletely characterized for blood clams subjected to a defined Cd exposure schedule.

We hypothesized that Cd-exposed blood clams would develop tissue-dependent metal burdens and redox trajectories, accompanied by changes in gill and hepatopancreatic architecture. To examine these relationships, we measured oxygen consumption, tissue Cd, antioxidant endpoints and histology during exposure to a nominal Cd concentration of 100 μg/L, with sampling up to 480 h. A separate experiment described hepatopancreatic expression profiles at the 0 h baseline and after 24 h and 21 d, supplemented by qRT-PCR measurements of selected targets. The study combines these observations to characterize tissue and temporal patterns and identify molecular candidates for subsequent functional investigation.

## 2. Materials and Methods

### 2.1. Experimental Animals and Acclimation

Adult blood clams were obtained from Datang, Bohai Bay, Tianjin, China, and identified from shell and external morphological features. Mean shell length was 42.6 ± 3.8 mm and mean whole-animal wet weight was 13.5 ± 2.4 g. Sex could not be determined because the gonads were undeveloped at sampling. Clams were acclimated for 7 d in aerated artificial seawater filtered through a 0.45 μm membrane, at 13 ± 1 °C, pH 7.8–8.0 and salinity 28–30 under natural light. Animals were randomly allocated to tanks, and tank positions were rotated during the experiment.

During acclimation and the low-concentration exposures, clams were fed concentrated Chlorella suspension approximately every 48 h, simultaneously with water renewal. The dry algal mass supplied at each feeding was equivalent to 4% of the shell-inclusive wet mass of the clams. Dissolved oxygen and ammonia nitrogen were measured before water renewal and were ≥10 mg/L and ≤0.1 mg/L, respectively.

### 2.2. Exposure and Sampling

Cadmium chloride hemipentahydrate (CdCl_2_·2.5H_2_O; analytical grade; Sinopharm, Beijing, China) was used to prepare the exposure solutions. Concentrations are expressed as nominal elemental Cd equivalents. The main concentration, 100 μg/L (0.1 mg/L), was selected with reference to previous Cd-exposure studies and the acute LC_50_ determined in this study.

The main experiment comprised control and Cd-exposed groups, each with three 100 L containers holding approximately 80 L of water and initially 45 clams per tank. Samples were collected at 0, 24, 96, 168 and 480 h. Water was replaced completely approximately every 48 h, followed by re-addition of Cd to the exposure group to restore the nominal concentration. No mortality occurred during the low-concentration chronic exposure.

At each time, three clams were sampled from each tank, giving nine tissue-sampled clams per treatment. Separate portions from these animals were used for biochemical assays, tissue-Cd measurements and histology. Biochemical assays were performed on individual animals. An additional three clams per tank were measured individually for oxygen consumption and removed afterwards. Respiration and biochemical assays included concurrent controls at each time; tissue-Cd and histological series used healthy 0 h animals as the baseline.

**Figure 1:**
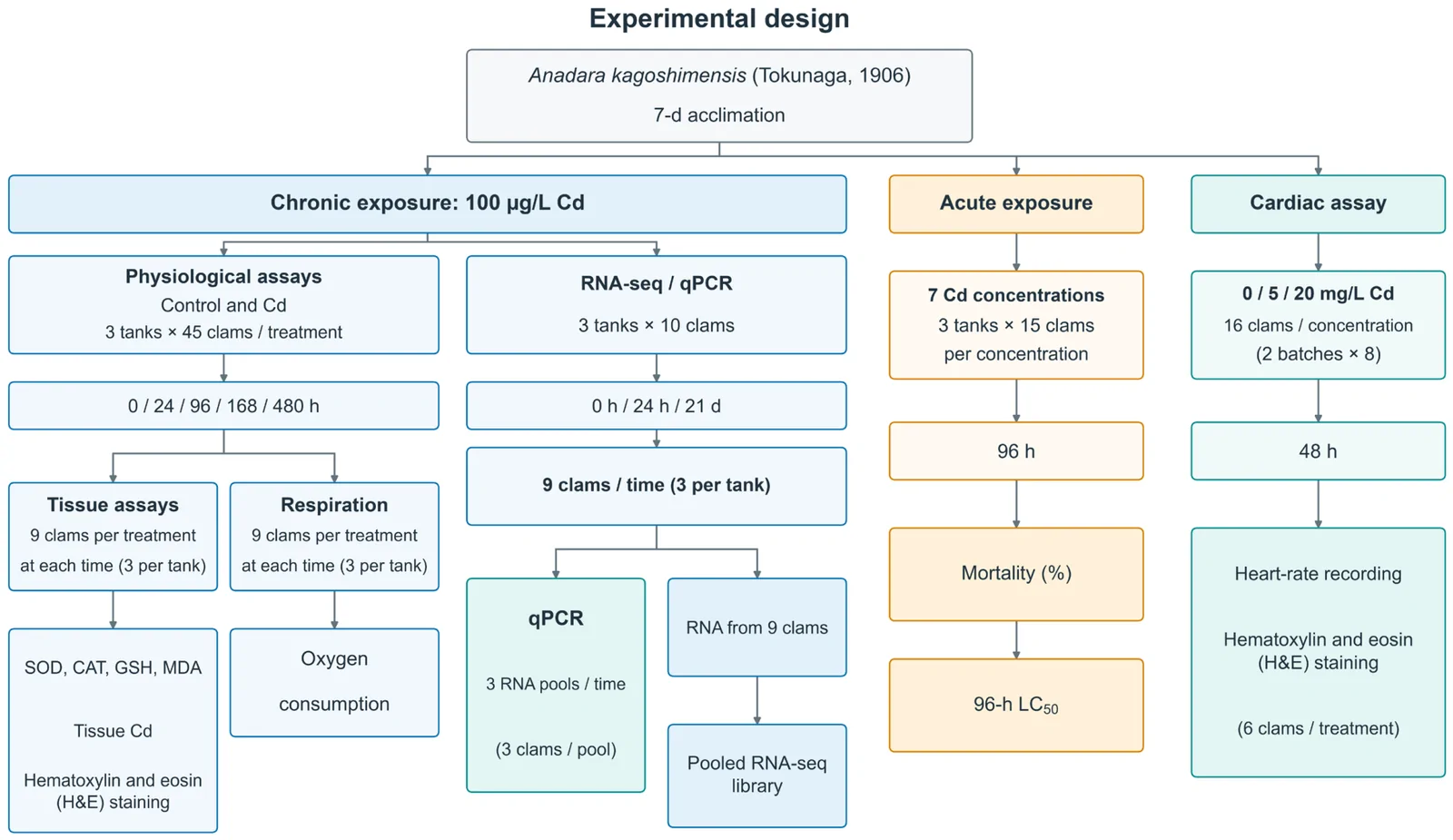
Experimental design and sample allocation. Physiological and tissue assays and RNA sampling were conducted at 100 μg/L Cd. At each RNA sampling time, three clams from each tank supplied three tank-specific RNA samples for qPCR; RNA from all nine clams was combined into one sequencing library. Separate animals were used for the acute toxicity and cardiac assays. Numbers and sampling times are given within each branch.

Three additional experimental components examined hepatopancreas RNA, acute mortality and cardiac responses. Their allocation and sampling, including the RNA aliquots used for sequencing and qRT-PCR, are shown in Figure 1.

### 2.3. Physiological, Chemical and Histological Analyses

#### 2.3.1. Oxygen Consumption

Nine clams per treatment and time, three from each tank, were placed individually in sealed 4 L respirometry chambers for 120 min. Oxygen consumption was calculated from the decline in dissolved oxygen after subtraction of background oxygen consumption and normalized to shell-inclusive wet weight (mg O_2_ h^−1^ g^−1^ wet weight). Different animals were measured at successive sampling times.

#### 2.3.2. Cadmium Accumulation

Gill and hepatopancreas tissues from the nine tissue-sampled clams were rinsed with filtered seawater, oven-dried at 80 °C for 12 h and digested in concentrated nitric acid. Mantle, muscle and blood were also examined at 480 h. Cd concentrations were measured by an external analytical platform using an Agilent 7500 inductively coupled plasma mass spectrometer (ICP-MS; Agilent Technologies, Santa Clara, CA, USA), with routine analytical quality control. Results are expressed as μg/g dry weight.

#### 2.3.3. Histopathology

Gill and hepatopancreas portions from nine clams per condition and time were fixed in Bouin’s solution for 24 h, dehydrated, embedded in paraffin, sectioned at 5 μm and stained with hematoxylin and eosin (H&E). Five sections were prepared for each tissue from each animal, and one was randomly selected for imaging. Section selection and histological assessment were performed without knowledge of treatment identity. Gill epithelial integrity, filament organization, digestive tubule architecture and cellular changes were examined qualitatively. 

In the 48 h cardiac assay, heart tissue from six living clams per concentration was processed for H&E histology using the same preparation procedure (Figure S5).

#### 2.3.4. Antioxidant and Lipid Peroxidation Biomarkers

Gill and hepatopancreas samples were assayed individually using Solarbio kits (Beijing, China): BC5165 for SOD, BC0205 for CAT, BC1170 for GSH, BC0025 for MDA and PC0020 for protein. GSH was measured in cuvettes (manufacturer-specified linear range, 3.125–250 μg/mL); SOD, CAT, MDA and protein assays were performed in 96-well plates. Tissue preparation followed the instructions supplied with the respective kits. SOD activity was measured by inhibition of WST-1 reduction at 450 nm, CAT activity by H_2_O_2_ decomposition at 240 nm, GSH content by DTNB colorimetry at 412 nm, and MDA content by the thiobarbituric acid reaction at 532 nm with correction at 600 nm. Protein concentration was determined by the bicinchoninic acid (BCA) method at 562 nm. Each sample was measured in three technical replicates for each assay. SOD and CAT activities are expressed as U/mg wet weight, GSH content as mg/g protein and MDA content as nmol/mg protein.

### 2.4. Hepatopancreas RNA-Seq and qRT-PCR

#### 2.4.1. RNA Sampling and Library Design

The RNA experiment used three 30 L tanks, each containing 24 L of water and initially ten clams. From each tank, three clams were randomly sampled before exposure (0 h) and after 24 h and 21 d at nominal 100 μg/L Cd. Hepatopancreas samples were frozen in liquid nitrogen and stored at −80 °C. Total RNA was extracted using TRIzol reagent (Invitrogen, Waltham, MA, USA), and quality was assessed by agarose gel electrophoresis, NanoDrop spectrophotometry and an Agilent 2100 Bioanalyzer (Agilent Technologies, Santa Clara, CA, USA). Husbandry followed Section 2.1.

For RNA-seq, RNA from nine clams (three per tank) was pooled at each sampling time. Three tank-specific RNA samples, each comprising three clams, were retained for separate reverse transcription and qRT-PCR.

#### 2.4.2. Sequencing and Bioinformatic Analysis

Poly(A)-enriched libraries were sequenced by Novogene (Beijing, China) using paired-end 150 bp reads on an Illumina NovaSeq 6000 platform (Illumina, Inc., San Diego, CA, USA). After adapter and low-quality read removal, transcripts were assembled de novo with Trinity v2.4.0 (minimum k-mer coverage, 3), clustered with Corset v1.09 [15] and quantified with RSEM v1.2.15. Functional annotations were assigned using Gene Ontology (GO) and the Kyoto Encyclopedia of Genes and Genomes (KEGG). The resulting assembly feature identifiers were used to join the expression and annotation tables.

Expression profiles were compared descriptively using fragments per kilobase of transcript per million mapped reads (FPKM). For each exposed-time versus 0 h comparison, features with FPKM ≥ 1 in at least one sample were retained. When both values were positive, log_2_ fold change was calculated as log_2_(FPKM_exposed/FPKM_0h); ratios ≥2 or ≤0.5 defined the higher- and lower-expression categories. Features detected in one sample alone were listed separately. GO and KEGG summaries report the numbers of annotated features in these expression categories. The complete three-library matrix and category counts are provided in Supplementary Data S1 and S2.

Table S1 gives the sequencing quality metrics for each library. In the selected-gene heatmaps, columns C, Cd_24h and Cd_21d represent the individual 0 h, 24 h and 21 d libraries. Expression was scaled from the minimum to the maximum within each row to show the relative profile across the three times. Rows are grouped by their functional annotations.

#### 2.4.3. qRT-PCR Assessment of Selected Genes

Ten targets were assessed by quantitative reverse-transcription PCR (qRT-PCR): BAX, CASP7, MT, SOD, TLR, TNN, TPM, NKA, KCN and HCN. Three tank-specific RNA samples per time, each derived from three clams, were separately reverse-transcribed into cDNA. Reactions were run on a CFX96 Touch system (Bio-Rad Laboratories, Inc., Hercules, CA, USA) using TB Green Premix Ex Taq II (Takara Bio Inc., Kusatsu, Shiga, Japan), with technical replicate reactions. Relative expression was calculated by the 2^−ΔΔCt^ method, using the geometric mean of β-actin and GAPDH expression for normalization and the 0 h samples as the calibrator [16].

Melting curves, product specificity, no-template controls, no-reverse-transcription controls and amplification efficiencies were checked. Primer sequences, amplicon lengths and efficiencies are listed in Table S2. The comparison with RNA-seq comprised ten targets at two exposure times.

### 2.5. Acute Mortality and Separate Cardiac Assay

The 96 h acute mortality experiment used nominal Cd concentrations of 0, 0.6, 1.1, 2.0, 3.6, 6.6 and 12 mg/L. Each concentration comprised three 30 L tanks containing approximately 24 L of water and 15 clams per tank. Water was renewed approximately every two days, followed by re-addition of Cd to the nominal concentration. Clams were not fed, and mortality was recorded daily. Mortality percentages, corresponding total death counts and the probit LC_50_ estimate are given in Table S4.

The 48 h cardiac assay used nominal Cd concentrations of 0, 5 and 20 mg/L. Sixteen clams per concentration were fitted with infrared sensors and measured in two batches of eight in an instrument holding 30 L of water. Heart-rate signals were recorded using an AMP03 amplifier (Newshift Lda., Leiria, Portugal), a PowerLab acquisition system and LabChart v7, following Liang et al. [17]. During recording, animals were not fed and water was not renewed. One representative individual trace per concentration is shown in Figure S4. Six living animals per concentration supplied heart tissue at the end of the assay (Figure S5). The two Cd doses were selected with reference to the acute mortality result.

### 2.6. Statistical Analysis

Data are summarized as means ± standard deviations (SD). Waterborne treatments were allocated to tanks; the main experiment used three tanks per treatment and sampled three animals per tank at each time. Physiological and biochemical summaries describe the nine individual measurements. The one-way analysis of variance (ANOVA) and Tukey honestly significant difference (HSD) comparisons shown in the figures were performed in SPSS v27.0 on the individual measurements. These individual-level analyses did not include tank as a model term.

Figure legends identify the comparisons represented by letters and asterisks. Time comparisons were made within each treatment or tissue, and between-treatment comparisons were made at each sampling time. RNA-seq results are reported as expression profiles and fold-change categories. qRT-PCR means and SD describe three tank-specific biological RNA samples per time; the regression summarizes agreement across the 20 selected target–time combinations.

Acute mortality percentages are summarized across three tanks of 15 clams per concentration. Total deaths were calculated from the reported mean percentages and the initial 45 clams per concentration. The 96 h LC_50_ and its 95% confidence interval were estimated by probit analysis. Cardiac traces and tissue sections are presented descriptively.

### 2.7. Use of Generative Artificial Intelligence

ChatGPT 5.6 and Codex 26.924 assisted with language polishing. The authors reviewed the outputs and take responsibility for the manuscript.

## 3. Results

### 3.1. Oxygen Consumption and Tissue Cd Burdens

**Figure 2:**
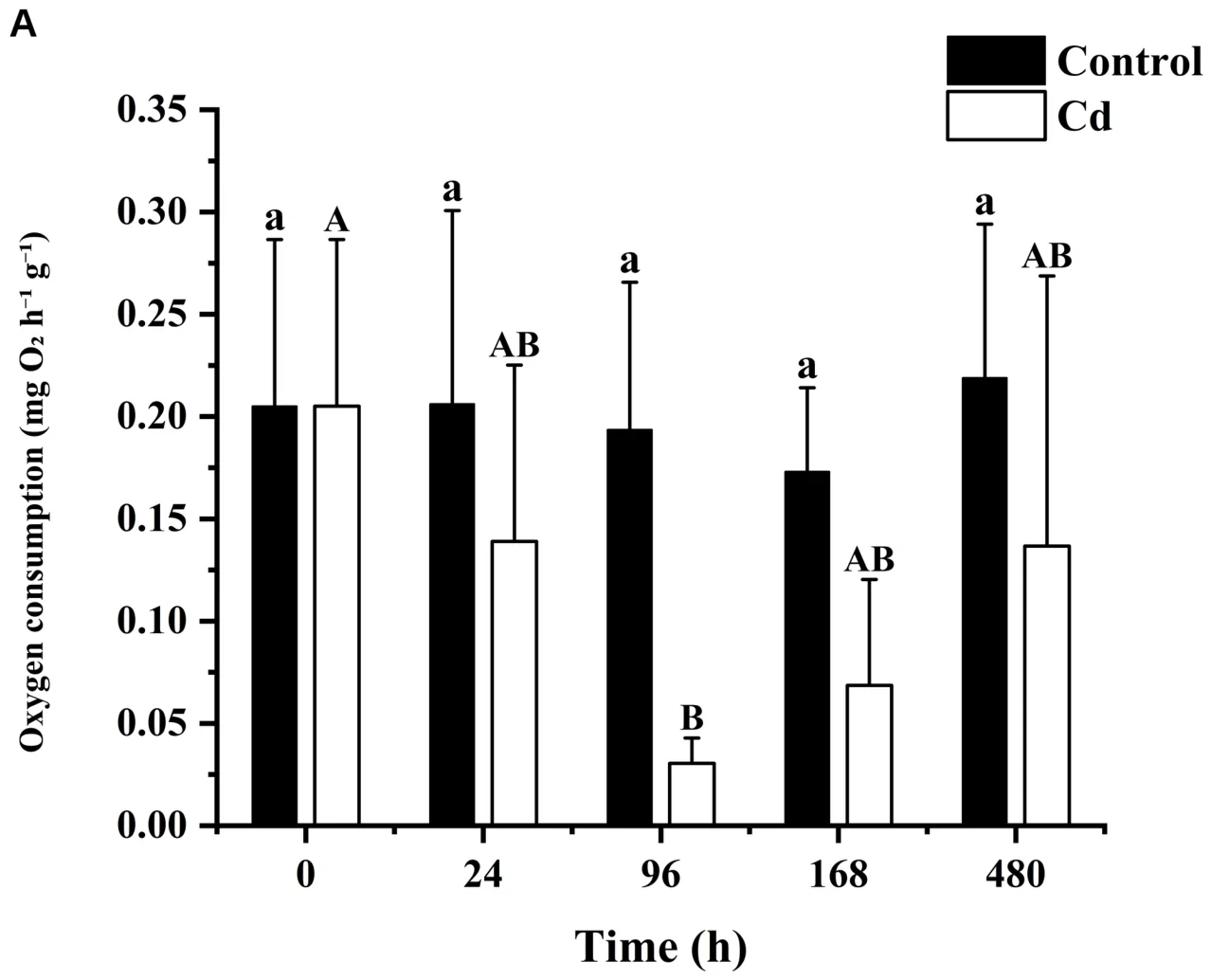

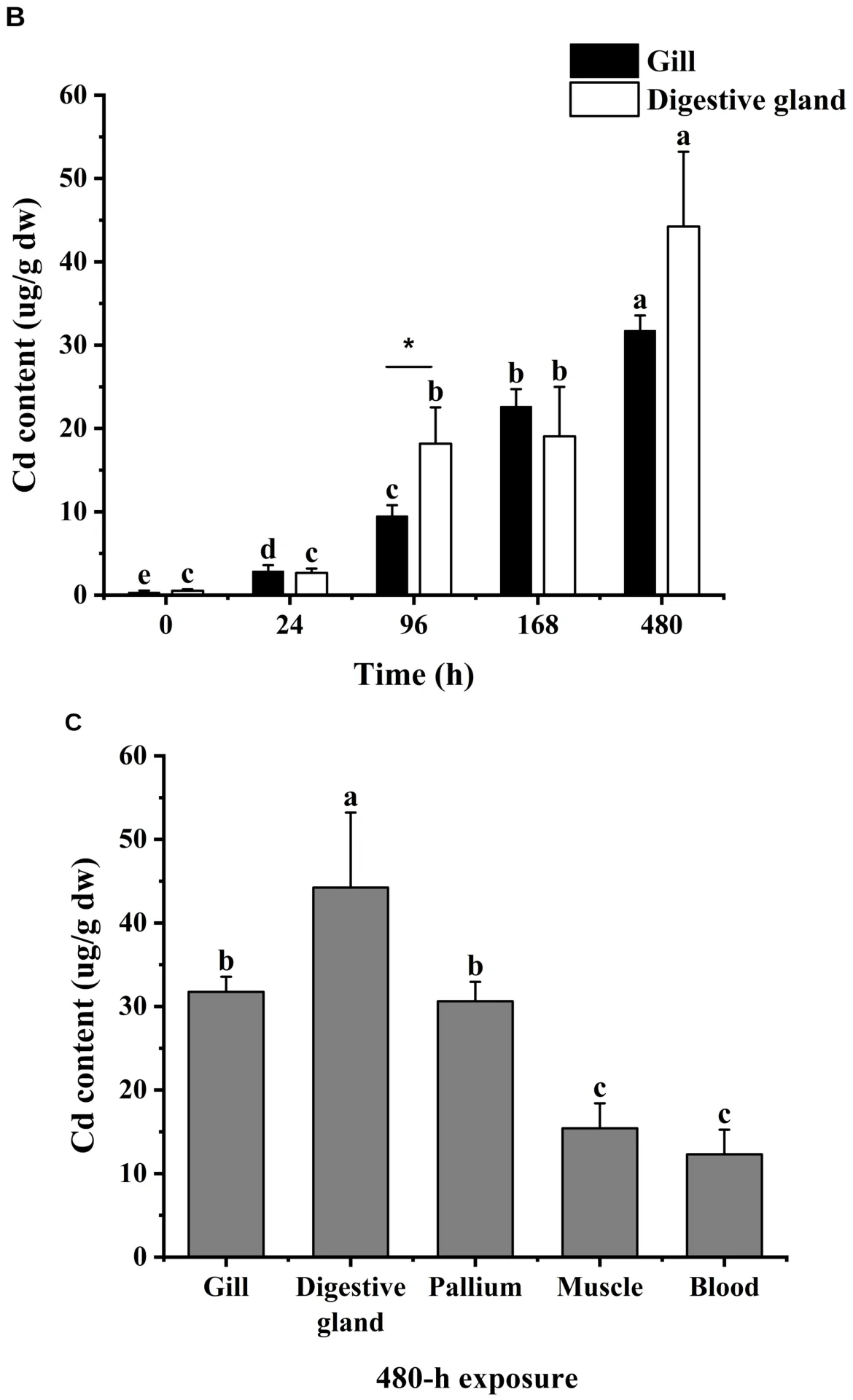
Oxygen consumption and tissue Cd concentrations during nominal 100 μg/L Cd exposure. (**A**) Oxygen consumption at 0, 24, 96, 168 and 480 h. (**B**) Gill and hepatopancreas Cd concentrations, with 0 h as the baseline. (**C**) Tissue Cd concentrations at 480 h. Digestive gland denotes hepatopancreas; pallium denotes mantle. Values are means ± SD from nine clams per treatment/time or sampling condition, comprising three animals from each of three tanks. In (**A**), lowercase letters indicate comparisons among times within the control group, and uppercase letters indicate comparisons among times within the Cd-exposed group. In (**B**), lowercase letters indicate comparisons among times within each tissue; in (**C**), lowercase letters indicate comparisons among tissues. Within each comparison set, groups sharing no letter differ significantly (individual-level one-way ANOVA followed by Tukey’s HSD test, *p* < 0.05). The asterisk in (**B**) denotes a significant difference between the two tissues at 96 h (*p* < 0.05).

Oxygen consumption in Cd-exposed clams declined from 24 to 96 h, reaching 0.031 ± 0.012 mg O_2_ h^−1^ g^−1^ wet weight at 96 h (Figure 2A). Exposed means subsequently increased at 168 and 480 h but remained below the corresponding control means. The control series showed a flatter temporal profile.

Gill and hepatopancreas Cd concentrations were low at 0 and 24 h and increased at later sampling times (Figure 2B). The hepatopancreas mean exceeded the gill mean at 96 h, whereas the gill mean was higher at 168 h. At 480 h, concentrations reached 44.23 ± 8.99 μg/g dry weight in the hepatopancreas and 31.75 ± 1.81 μg/g in the gill.

At 480 h, mean Cd concentrations decreased in the order hepatopancreas, gill, mantle, muscle and blood (Figure 2C).

### 3.2. Tissue-Specific Redox Responses

**Figure 3:**
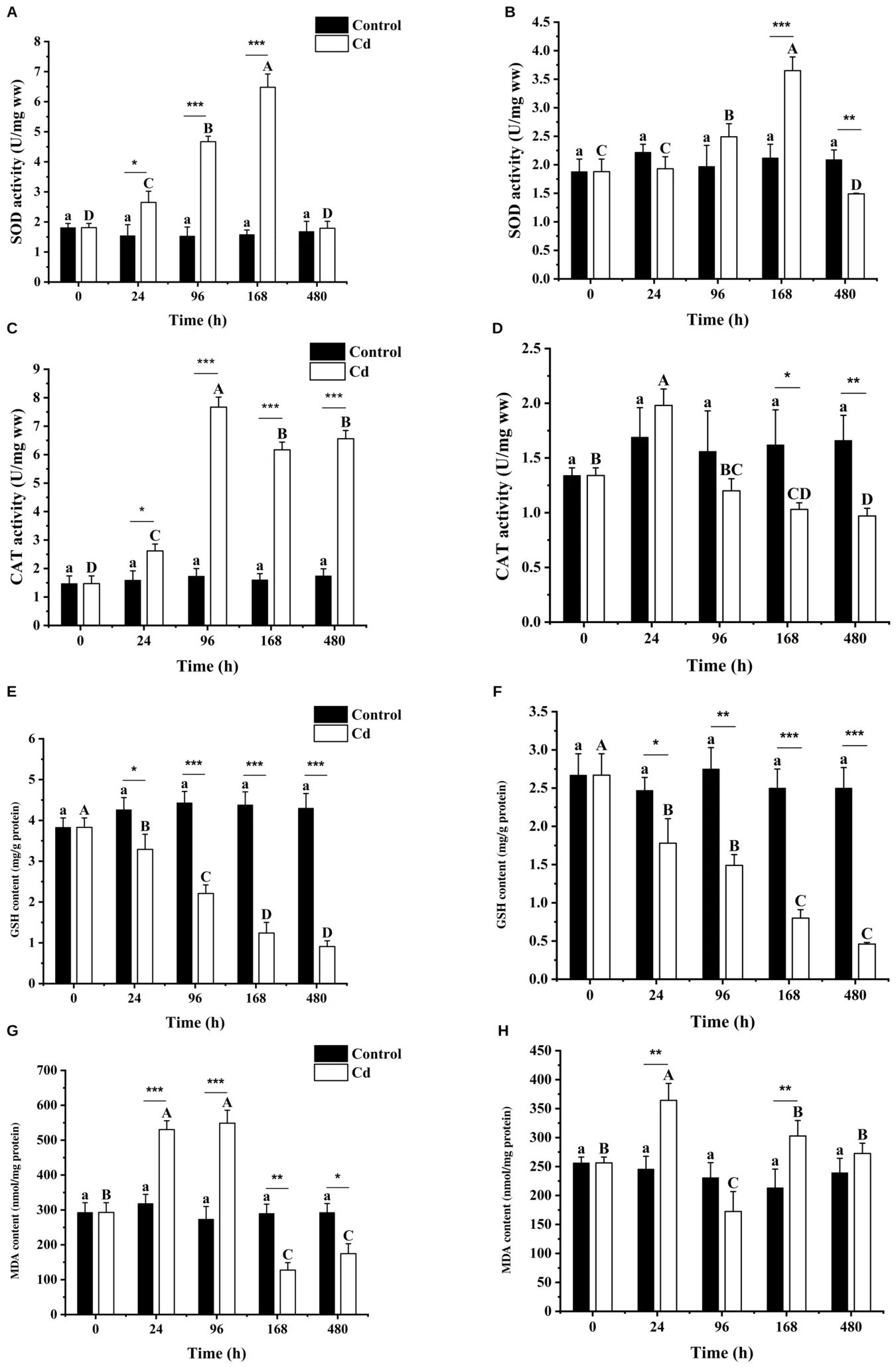
Redox biomarkers in the hepatopancreas (**left column**) and gill (**right column**). Panels show SOD activity (**A**,**B**), CAT activity (**C**,**D**), GSH content (**E**,**F**) and MDA content (**G**,**H**). Values are means ± SD for nine individual clams per treatment and time, sampled as three animals from each of three tanks. SOD and CAT are expressed as U/mg wet weight, GSH as mg/g protein and MDA as nmol/mg protein. Uppercase and lowercase letters indicate individual-level one-way ANOVA/Tukey comparisons among times within the Cd and control groups, respectively; different letters denote *p* < 0.05. Asterisks compare treatments at the indicated time: * *p* < 0.05, ** *p* < 0.01 and *** *p* < 0.001. GSH and MDA contents (**E**–**H**).

Hepatopancreas and gill SOD activities reached their highest means at 168 h, at 6.48 ± 0.44 and 3.65 ± 0.24 U/mg wet weight, respectively (Figure 3A,B). Hepatopancreas CAT peaked earlier at 96 h (7.67 ± 0.35 U/mg wet weight), whereas gill CAT reached an early maximum at 24 h and subsequently declined below the corresponding control means at later times (Figure 3C,D).

GSH content declined from 24 to 480 h in both tissues (Figure 3E,F). At 480 h, the exposed values were 0.91 ± 0.14 mg/g protein in the hepatopancreas and 0.46 ± 0.02 mg/g protein in the gill. The corresponding control series did not show the same sustained decline.

MDA content changed non-monotonically (Figure 3G,H). Hepatopancreas means were above the controls at 24 and 96 h and below them at 168 and 480 h. Gill MDA showed an early maximum at 24 h, a lower value at 96 h and a second maximum at 168 h, followed by a decrease at 480 h.

### 3.3. Gill and Hepatopancreas Histology

**Figure 4:**
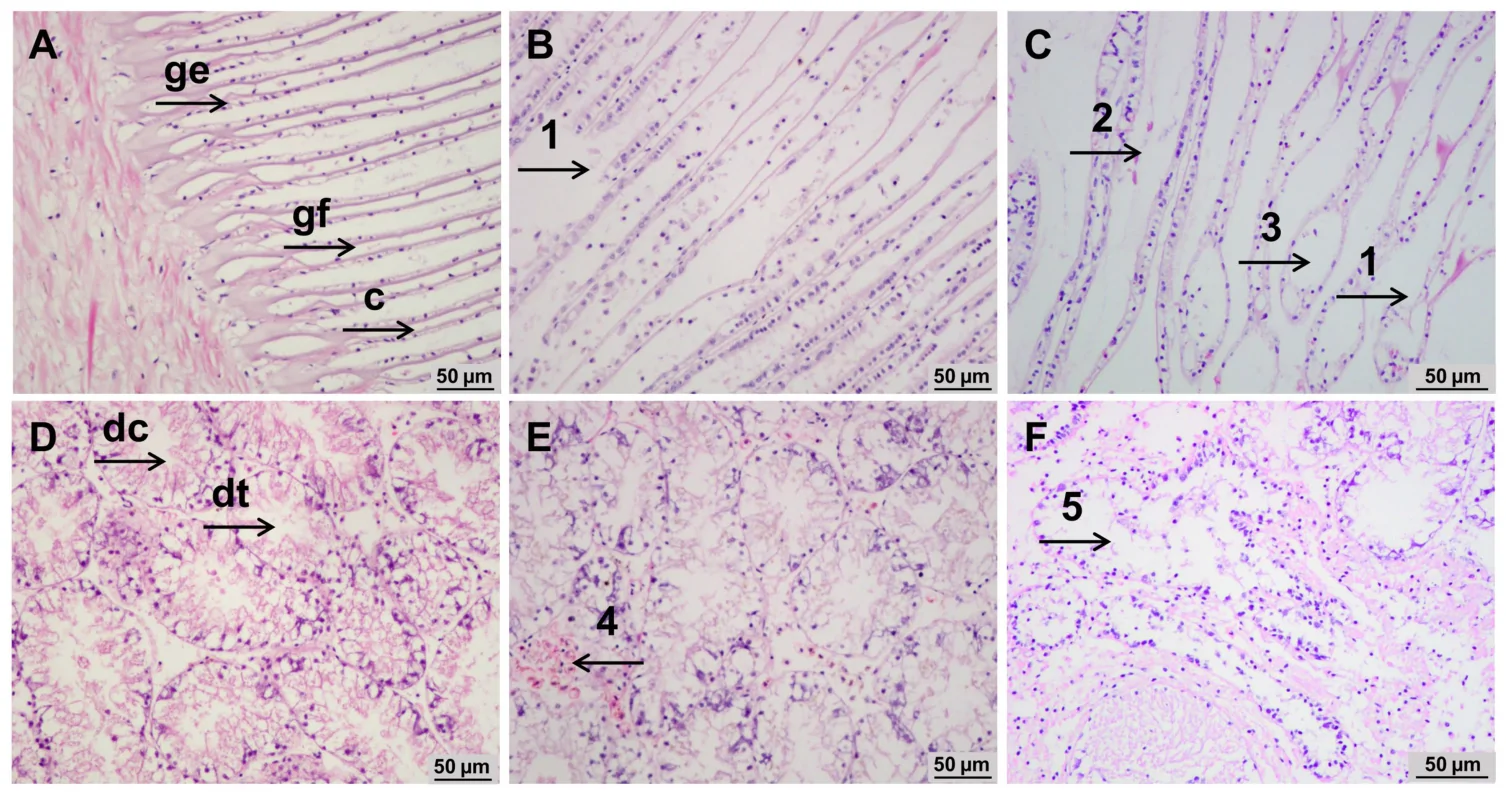
Representative hematoxylin and eosin (H&E)-stained sections of the gill and hepatopancreas. (**A**–**C**) show gill sections at 0 h, 96 h and 480 h; (**D**–**F**) show hepatopancreas sections at the corresponding times. Tissues were sampled from nine clams per condition and time, three from each tank. Five sections per tissue per animal were prepared, one was randomly selected for imaging, and section selection and assessment were blinded to treatment. Abbreviations: ge, gill epithelium; gf, gill filament; c, cilia; dc, digestive cells; dt, digestive tubule. Arrows mark epithelial detachment (1), hemocyte infiltration (2, 4) and cellular disintegration (3, 5). Scale bars are shown in the panels. The full time series is provided in Figure S1.

The 0 h gill sections showed closely arranged filaments and a continuous epithelial outline. At 96 and 480 h, sections showed epithelial detachment, irregular filament profiles and focal hemocyte infiltration (Figure 4A–C).

The baseline hepatopancreas had well-defined digestive tubules and cellular outlines. Exposed samples showed irregular tubule architecture, detached cellular material, cellular disintegration and hemocyte infiltration (Figure 4D–F). The additional 24 h and 168 h sections are shown in Figure S1, and the principal morphological observations are summarized in Table 1.

In the cardiac assay, the representative exposed traces showed lower beat frequencies than the displayed control trace (Figure S4). Terminal heart sections showed changes in fiber organization, interstitial spaces and cellular infiltration at the higher Cd concentrations (Figure S5).

### 3.4. Sequencing Quality and Expression Profiles

The three libraries yielded 20.9–22.0 million raw read pairs and 20.6–21.5 million clean read pairs each, corresponding to 6.17–6.46 Gb of clean sequence per library (Table S1). Q20 values were 97.39–97.49%, Q30 values were 92.62–92.85%, and GC content was 35.70–36.74%. The expression matrix contained 129,639 assembly features. The assembly contained 369,091 transcripts (N50, 1128 bp) and 129,639 unigenes (N50, 1132 bp). BUSCO assessment of the unigene set classified 828 orthologues as complete single-copy, 17 as complete duplicated, 113 as fragmented and 20 as missing.

**Figure 5:**
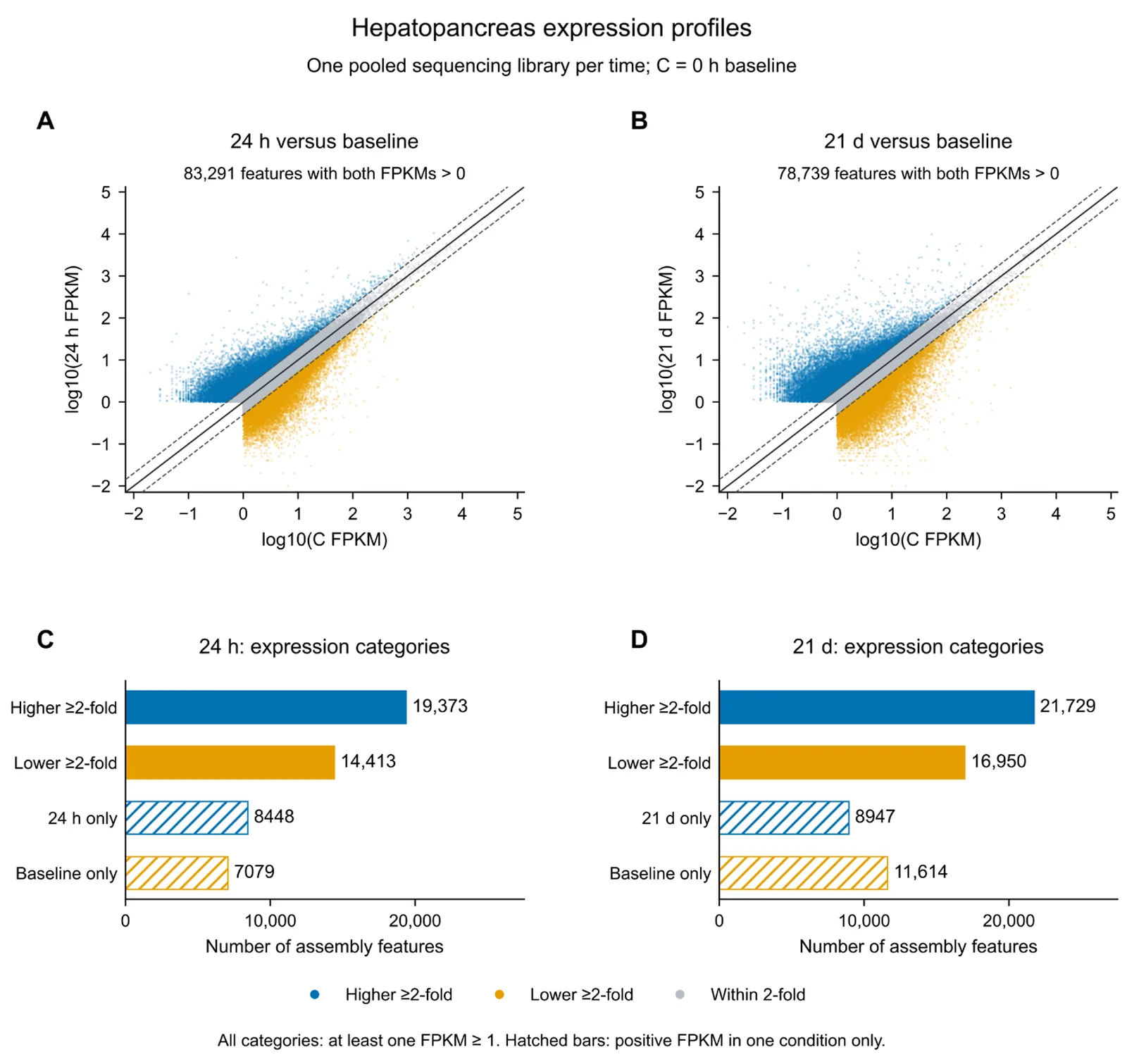
Hepatopancreas expression profiles in the three pooled RNA-seq libraries. (**A**,**B**) compare 24 h and 21 d with the 0 h baseline (0 h). Scatterplots include features with positive FPKM in both samples and FPKM ≥ 1 in at least one. The solid line indicates equal expression, and dashed lines indicate twofold differences. (**C**,**D**) summarize features with at least twofold higher or lower expression and features detected in one sample alone. Fold changes are oriented as exposed sample/0 h. Each library contains RNA pooled from nine animals sampled across three tanks. These are descriptive expression categories.

Among features with positive expression in both samples and FPKM ≥1 in at least one, 19,373 had at least twofold higher expression and 14,413 had at least twofold lower expression at 24 h relative to 0 h. At 21 d, the corresponding counts were 21,729 and 16,950 (Figure 5). A further 8448 and 8947 features were detected only in the respective exposed samples, while 7079 and 11,614 were detected only in the corresponding baseline comparisons.

Selected KEGG categories contained different combinations of higher- and lower-expression features (Table S3). At 24 h, fat digestion and absorption contained 34 higher and 5 lower features, glycerolipid metabolism 33 and 2, and pancreatic secretion 31 and 8. At 21 d, the corresponding pairs were 24 and 14, 15 and 15, and 28 and 12.

Lysosome-associated features included 89 higher and 11 lower features at 24 h and 63 higher and 62 lower features at 21 d. Antigen processing and presentation contained 17 higher and 4 lower features at 24 h and 19 higher and 17 lower features at 21 d. The complete GO and KEGG category counts are provided in Supplementary Data S2.

Thus, the later profile contained more twofold expression differences overall, while the direction within individual functional categories remained heterogeneous.

### 3.5. Cell-Death and Contractile-Associated Expression Profiles

**Figure 6:**
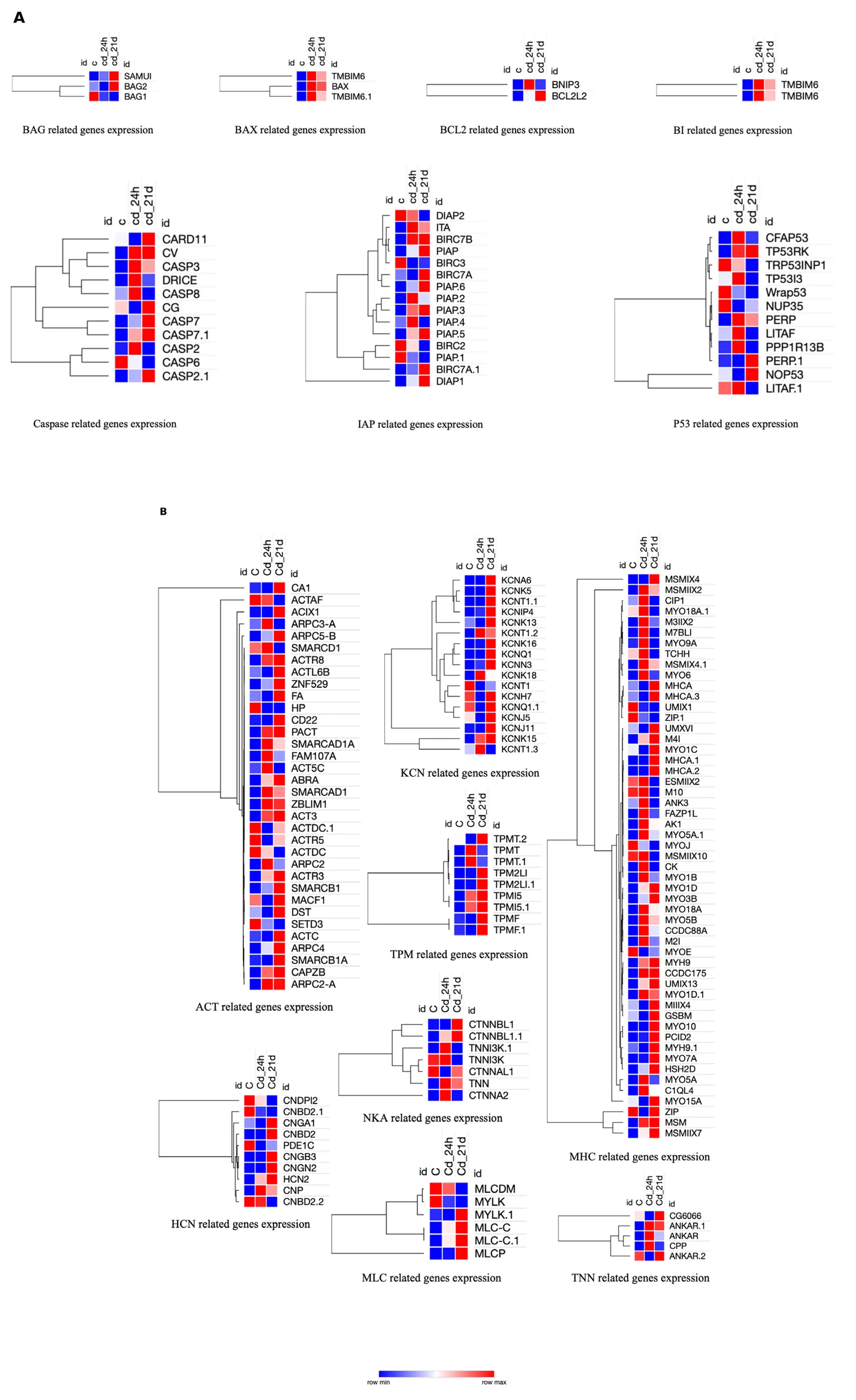
Hepatopancreas expression heatmaps for cell-death and contractile-associated annotations. (**A**) BAG, BAX, BCL2, BI, caspase, IAP and p53 groups. (**B**) ACT, KCN, TPM, NKA, MLC, HCN, TNN and MHC groups. Columns C, Cd_24h and Cd_21d represent the individual pooled libraries at 0 h, 24 h and 21 d. Expression is scaled from minimum to maximum within each row. Expression values are scaled within each row from the row minimum (blue) to the row maximum (red), with white representing intermediate values. Deeper blue and red shades indicate lower and higher relative expression, respectively, within that row. Rows and group headings follow the transcript annotations.

Heatmaps showed different profiles within the BAG, BAX, BCL2, BI, caspase, IAP and p53 annotation groups (Figure 6A). Several caspase-labeled rows were relatively higher in the exposed samples than at 0 h. Other rows reached their highest relative values at different times, including contrasting patterns within the IAP and p53 groups.

Contractile, cytoskeletal and ion transport annotations also showed distinct temporal profiles (Figure 6B). Many KCN-, TPM- and MHC-labeled rows had higher relative expression at 21 d, while others peaked at 0 or 24 h. NKA and HCN groups contained mixed profiles. Antioxidant- and immune-associated heatmaps are shown in Figure S2 and Figure S3.

### 3.6. Metal Handling and Redox-Associated Expression Profiles

**Figure 7:**
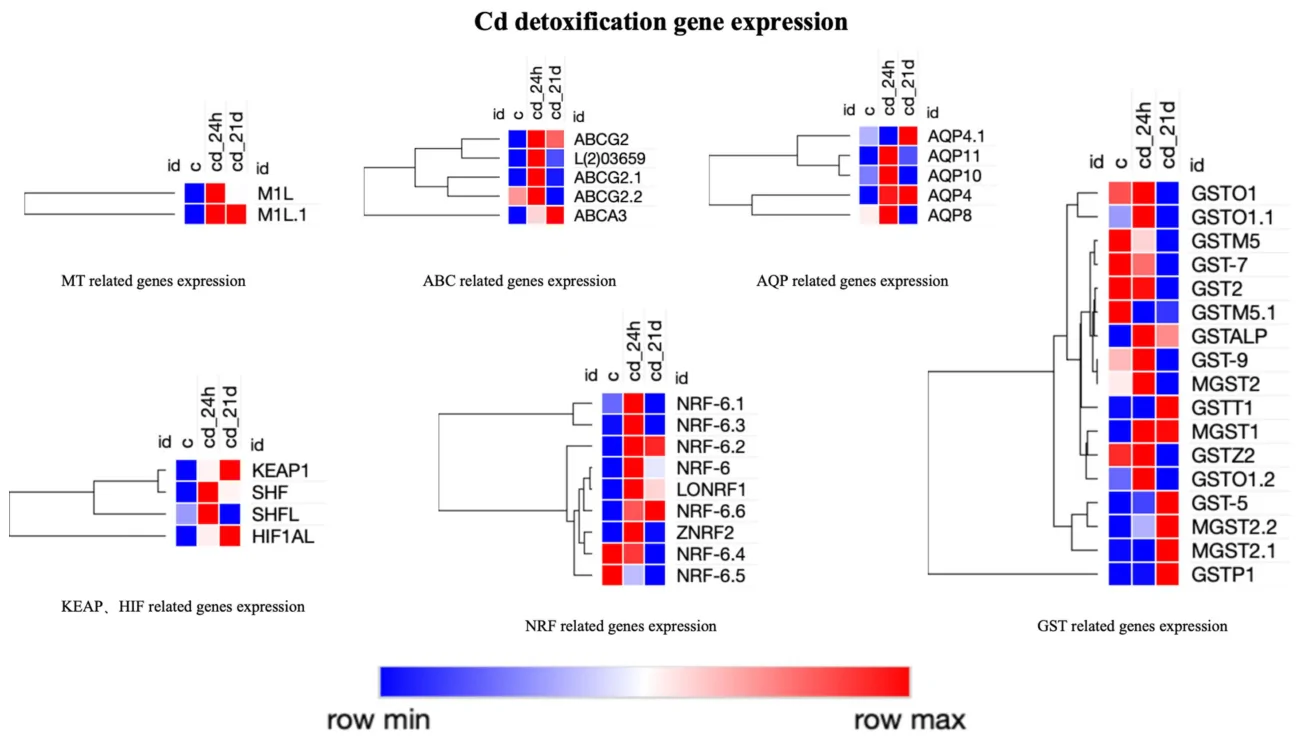
Hepatopancreas expression heatmaps for MT, ABC, AQP, KEAP/HIF, NRF and GST annotation groups. Columns show the individual pooled C, Cd_24h and Cd_21d libraries. Colors represent minimum-to-maximum scaling within each row, as in Figure 6.

MT-associated rows showed higher relative expression in exposed samples, with differences in the magnitude and later pattern between rows (Figure 7). Several ABC- and AQP-labeled rows peaked at 24 h, whereas other rows followed different trajectories. KEAP/HIF, NRF and GST groups included both early and late maxima.

### 3.7. Selected-Target qRT-PCR Comparison

**Figure 8:**
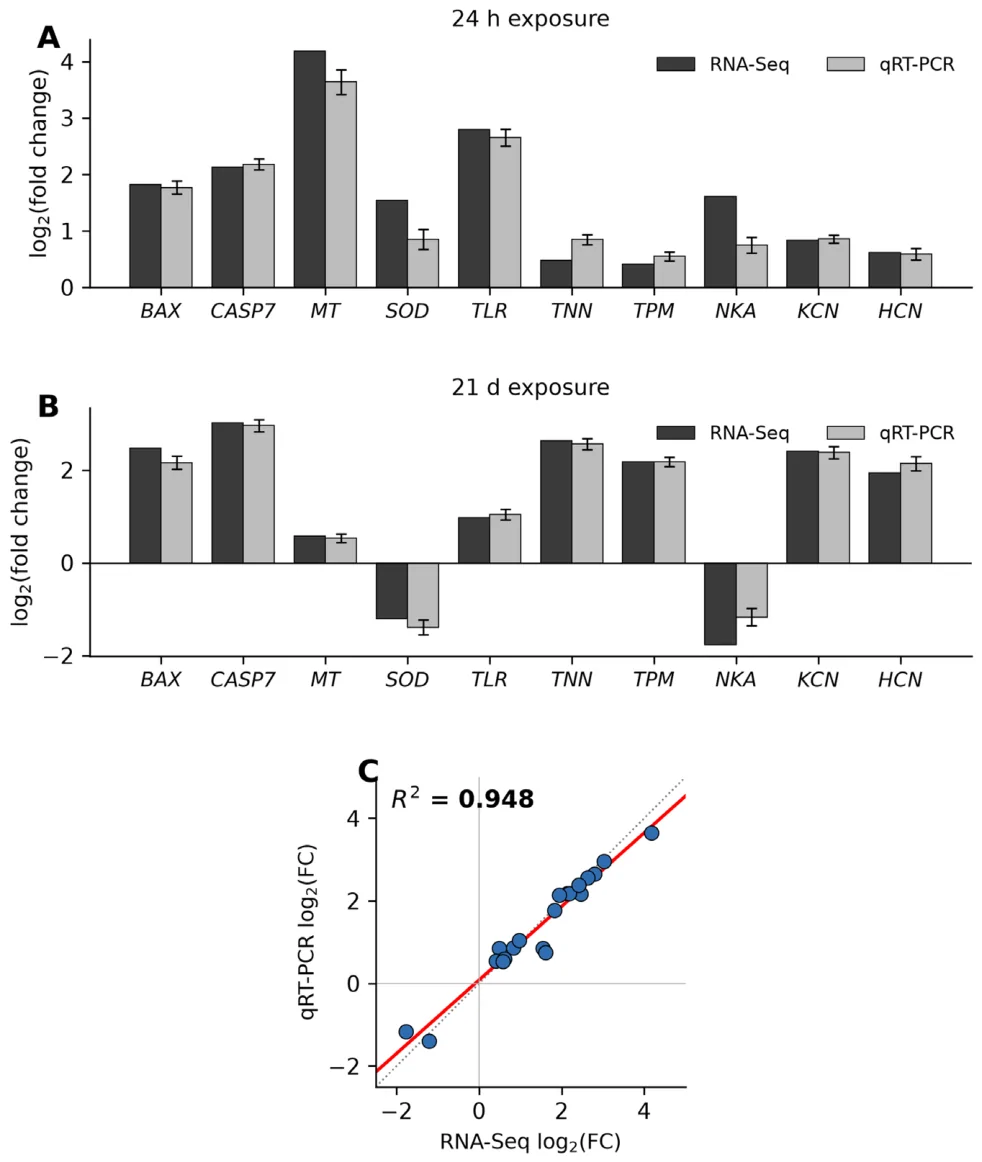
Comparison of RNA-seq and qRT-PCR expression estimates for ten targets. (**A**,**B**) show log_2_ fold changes at 24 h and 21 d relative to 0 h. qRT-PCR values are means ± SD from three tank-specific biological RNA samples per time, each comprising three clams, normalized to the geometric mean of β-actin and GAPDH. The sequencing library at each time combined RNA from the same nine animals. (**C**) shows the descriptive regression across 20 target–time combinations (R^2^ = 0.948).

The qRT-PCR profiles of the ten selected targets followed the directions shown in the RNA-seq comparison (Figure 8A,B). MT had the largest positive change among these targets at 24 h. At 21 d, SOD and NKA showed negative changes, while BAX, CASP7, TLR, TNN, TPM, KCN and HCN remained positive.

Across the 20 target–time combinations, the comparison gave R^2^ = 0.948 (Figure 8C).

### 3.8. Acute Mortality

Mortality in the 96 h acute test increased from 0% in the control to 86.67 ± 6.67% at 12 mg/L Cd (Table S4). The corresponding total deaths were 0 and 39 of the 45 clams assigned to each concentration. The 96 h LC_50_ was 3.708 mg/L (95% confidence interval, 2.780–5.213 mg/L).

## 4. Discussion

The principal pattern across the physiological and tissue measurements was a separation among the timing of Cd accumulation, antioxidant changes and respiratory minima. Higher tissue burdens at later sampling times accompanied different enzyme maxima in the gill and hepatopancreas, a sustained decline in GSH and non-monotonic MDA profiles. The tissue response is therefore better described as a set of interacting trajectories than as a uniform progression from antioxidant activation to damage. Histological observations add the corresponding structural context, while the separate hepatopancreatic RNA profiles identify candidate functions associated with the sampled conditions. Together, these measurements connect internal metal distribution with several aspects of animal condition and help identify the tissues and periods in which these relationships are most apparent.

The hepatopancreas contained the highest mean Cd concentration at 480 h, reaching 44.23 ± 8.99 μg/g dry weight compared with 31.75 ± 1.81 μg/g in the gill. This terminal distribution is consistent with the importance of the digestive tissues in internal metal handling and with tissue-dependent accumulation reported in other bivalves [5,6,7,18]. The ordering changed during exposure, however; the hepatopancreas mean exceeded the gill mean at 96 h, whereas the reverse occurred at 168 h. Uptake at exchange surfaces, redistribution and tissue-specific retention may contribute differently as exposure proceeds. A high terminal concentration consequently summarizes the accumulated outcome of these processes rather than their timing. Total tissue Cd also includes metal associated with different binding and storage compartments [19,20,21], making its relationship with biochemical condition more informative when interpreted alongside the tissue time series.

Oxygen consumption reached its lowest exposed mean at 96 h, 0.031 ± 0.012 mg O_2_ h^−1^ g^−1^ wet weight, during the increase in tissue Cd. Later exposed means rose but remained numerically below the corresponding controls. Bivalve respiration integrates valve opening, ventilation, activity and metabolic demand, all of which can change under environmental stress [22]. The observed minimum may therefore involve both the delivery of oxygen and its use by tissues. Its subsequent increase could reflect changes in ventilation or metabolic demand as exposure continued. The absence of a similarly pronounced minimum in controls provides useful physiological context. Measurements pairing oxygen consumption with valve behavior and tissue condition would help distinguish reduced exchange from altered metabolic demand and explain why the respiratory trajectory differs from the continuing increase in internal Cd.

The antioxidant enzymes showed distinct temporal relationships in the two tissues. Hepatopancreatic SOD reached 6.48 ± 0.44 U/mg wet weight at 168 h, when gill SOD also peaked at 3.65 ± 0.24 U/mg wet weight. Hepatopancreatic CAT reached its maximum of 7.67 ± 0.35 U/mg wet weight earlier, at 96 h, whereas gill CAT peaked at 24 h and subsequently declined. These differences are consistent with antioxidant components responding on different time scales [6,7,9]. GSH followed a more sustained downward trajectory, reaching 0.91 ± 0.14 and 0.46 ± 0.02 mg/g protein in the hepatopancreas and gill, respectively, at 480 h. Changes in its use, synthesis and regeneration offer possible explanations for this decline. Recent work in juvenile Manila clams likewise found asynchronous antioxidant responses to Cd and seawater acidification [23], emphasizing the dependence of redox profiles on both the endpoint and exposure conditions.

MDA added a different dimension to the redox response. Hepatopancreatic values exceeded the controls at 24 and 96 h but were lower at 168 and 480 h; gill values had maxima at 24 and 168 h separated by a lower 96 h value. This pattern indicates that lipid-peroxidation-associated measurements did not increase continuously with tissue Cd or mirror the GSH decline. Comparable endpoint separation was observed during intermittent Hg/Cd exposure in *Mytilus edulis*, where tissue accumulation and digestive gland changes occurred without increased TBARS [24]. Different exposure regimes can therefore produce different relationships among metal burden, tissue condition and oxidative markers. Cross-species comparisons also require attention to the endpoint: FTIR measurements in Cd/Pb-exposed *Carassius auratus gibelio* examined tissue structure and composition [25], while molluscan studies have assessed oxidative biomarkers under different metals and organic contaminants [26,27,28,29,30]. Assay denominators also matter: SOD and CAT were normalized to wet tissue mass, whereas GSH and MDA were normalized to protein. Interpretation should follow each endpoint’s trajectory, with tissue water and protein content influencing comparisons of magnitude.

The histological features give these biochemical profiles a tissue-level context. Epithelial detachment, irregular gill filaments and cellular disintegration can affect the organization of the exchange surface, while disrupted hepatopancreatic tubules concern a tissue involved in digestion and internal processing. In Cd-exposed *Meretrix meretrix*, altered gill architecture accompanied oxidative changes, with partial improvement of several endpoints after transfer to clean water [31]. The higher exposure concentration and depuration phase in that study distinguish its trajectory from the present experiment. Standardized histopathological frameworks combine the extent and importance of alterations, and fish-health studies have applied these principles to organ-level assessment [32,33,34]. Here, blinded interpretation of randomly selected sections supports a qualitative description of architecture. The shared animals used for tissue chemistry and histology provide complementary observations of condition at each sampling time.

The descriptive RNA comparisons extend the investigation to hepatopancreatic expression profiles. Among features with FPKM ≥ 1 in at least one condition and positive values in both members of a comparison, 19,373 were at least twofold higher and 14,413 at least twofold lower at 24 h than at baseline. The corresponding counts at 21 d were 21,729 and 16,950. Features detected only in one member of a comparison were kept in separate categories. The larger numbers at 21 d describe a broader departure from the sampled baseline under a common screening rule. Bulk RNA composition can vary with the physiological state and cellular composition of a tissue, so these profiles provide a starting point for examining candidate responses alongside the biochemical and morphological time series.

Functional annotation shows that digestive, lipid-processing, lysosomal and defense-related categories were represented at both sampling times. Within glycerolipid metabolism, the twofold candidates comprised 33 higher and two lower features at 24 h, compared with 15 in each direction at 21 d. Lysosome annotations included 89 higher and 11 lower features at 24 h and 63 higher and 62 lower features at 21 d. The changing balance within these categories is more informative than assigning an entire sampling time to one biological process. Fat digestion and absorption, pancreatic secretion, and antigen processing and presentation add further candidate associations (Table S3). These are annotation counts, with broad pathway names describing shared molecular functions. Cd studies in other clams and work on immunometabolism provide a rationale for investigating connections among nutrient processing, cellular turnover and defense [8,11,12,35].

The selected transcript heatmaps place these broad annotations beside more specific candidates. MT-associated rows were relatively higher in the exposed samples, while ABC-, AQP-, NRF- and GST-associated rows displayed different temporal profiles. Metallothioneins and essential-metal homeostasis are relevant to Cd handling [21,36], making these annotations useful starting points for examining the relationship between internal burden and redox condition. Cell-death-associated groups and cytoskeletal or ion transport annotations likewise connect with questions raised by altered tissue architecture. Molluscan hemocyte and receptor studies further support investigating defense-associated annotations in this context [37,38,39]. Each heatmap column represents one hepatopancreatic library, and within-row scaling emphasizes its relative position across the three samples. The displays therefore help compare candidate transcript patterns, while abundance comparisons among different rows require the underlying expression values. Contractile and channel annotations concern the hepatopancreas, where their possible cellular roles should be considered separately from the cardiac recordings.

The qRT-PCR measurements provide a focused comparison for ten annotations spanning metal handling, antioxidant defense, ion transport and cell-death-associated functions. Three tank-specific RNA samples were retained from the same nine clams used to construct each sequencing library and reverse-transcribed separately. The qRT-PCR samples preserve the tank-level biological sampling, while sequencing describes the combined RNA pool. Normalization used the geometric mean of β-actin and GAPDH. This complementary measurement adds target-level information to the expression profiles and helps prioritize candidates for functional characterization.

The acute mortality and cardiac assays define a separate, higher-concentration context. The 96 h LC_50_ of 3.708 mg/L summarizes the acute mortality response under the test conditions, while comparisons among bivalve studies show that such estimates depend on species and exposure regime [40,41]. Cardiac recording can complement other physiological measurements in molluscs [17,42,43,44,45]. Here, the selected exposed traces had lower beat frequencies than the displayed control trace, and sections from surviving animals showed differences in fiber organization and interstitial spaces. These observations arose at 5 and 20 mg/L, respectively 50 and 200 times the main concentration, during an assay without feeding or water renewal. Studies of calcium channels, cardiac gene expression and contractile proteins provide possible mechanisms for focused investigation [46,47,48]. The high-dose observations identify cardiac performance as a complementary endpoint for examining the physiological consequences of metal exposure.

The temporal patterns suggest specific connections worth examining within an integrated sampling design. The 96 h respiratory minimum coincided with rising tissue Cd, whereas later tissue burdens accompanied lower GSH content and a different balance of antioxidant activities. Hepatopancreatic expression profiles identify metal-binding, transport and cellular defense candidates that can be examined alongside these tissue measurements. Pairing valve behavior and respiration with tissue chemistry, quantitative histology and replicated molecular sampling would help resolve how the responses develop together. The present observations provide a tissue- and time-resolved basis for selecting those measurements and candidate targets.

## 5. Conclusions

Exposure to nominal 100 μg/L Cd was accompanied by tissue-dependent metal burdens, distinct antioxidant trajectories and qualitative changes in blood clam gill and hepatopancreatic architecture. The respiratory minimum at 96 h, sustained GSH decrease and non-monotonic MDA profiles show that these endpoints followed different time courses. Descriptive hepatopancreatic RNA comparisons identified baseline-relative expression differences and candidates associated with metal handling, metabolism and cellular defense, while qRT-PCR provided additional measurements for selected targets. Together, the observations define tissue and temporal profiles that can guide focused studies of the relationships between internal Cd, physiological condition and transcript expression.

## Figures and Tables

**Table 1:** Qualitative histological observations in the gill and hepatopancreas.

Tissue and Panels	Sampling Condition	Visible Features
Gill, Figure 4A	Healthy 0 h baseline	Closely arranged filaments and continuous epithelial outline
Gill, Figure 4B,C	96 and 480 h Cd exposure	Epithelial detachment, irregular filament profiles, and hemocyte infiltration
Hepatopancreas, Figure 4D	Healthy 0 h baseline	Recognizable digestive tubules and cellular outlines
Hepatopancreas, Figure 4E,F	96 and 480 h Cd exposure	Irregular tubule architecture, cellular disintegration, and hemocyte infiltration

## Data Availability

The raw RNA-seq reads have been submitted to the Genome Sequence Archive (GSA) under BioProject PRJCA075468. The three-library gene-expression matrix, descriptive fold-change categories and GO/KEGG annotation counts are provided in Supplementary Data S1 and S2. Sequencing quality metrics, primer information, mortality summaries and supplementary figures accompany the article.

## Supplementary Materials

The following supporting information can be downloaded at https://www.mdpi.com/article/10.3390/toxics14100883/s1, Table S1, library-level sequencing quality; Table S2, qRT-PCR primers and amplification efficiencies; Table S3, selected functional annotation counts; Table S4, acute mortality percentages and total counts; Figure S1, gill and hepatopancreas histological series; Figures S2 and S3, antioxidant- and immune-associated heatmaps; Figure S4, representative individual heart-rate traces; Figure S5, heart histology; Supplementary Data S1 provides the three-library expression matrix and fold-change categories; Supplementary Data S2 provides complete GO and KEGG annotation-category counts.
