# Supplementary material for: Physiological and Transcriptional Responses of the Blood Clam Anadara kagoshimensis (Tokunaga, 1906) During Cadmium Exposure
Source: Toxics. 2026 Sep 30;14(10):883. doi: 10.3390/toxics14100883 (PMC13709405; doi:10.3390/toxics14100883)
Supplement: Supplementary file 1 [file toxics-14-00883-s001.zip › toxics-4557668-supplementary.pdf]

Supplementary Materials

Physiological and transcriptional responses of the blood clam *Anadara kagoshimensis* (Tokunaga, 1906) during cadmium exposure

Supplementary methods

The main experiment used three tanks per treatment and nine individual tissue samples per treatment and time. Respiration was measured in an additional nine clams. The separate RNA experiment sampled nine animals at each of 0 h, 24 h and 21 d: three tank-specific RNA samples were retained for qRT-PCR, and RNA from all nine animals was combined into one sequencing library. Experimental allocation is shown in Figure 1 of the main text.

The acute mortality experiment used three tanks of 15 clams per concentration, with water renewal approximately every two days. The cardiac assay used 16 instrumented clams per concentration in two batches of eight, with a 30 L working water volume. Animals were not fed during either assay, and water was not renewed during cardiac recording. Six living clams per concentration supplied terminal heart sections.

Supplementary Tables

Table S1. Sequencing quality of the three pooled RNA-seq libraries.

Condition	Raw read pairs	Clean read pairs	**Clean bases**	**Error rate (%)**	**Q20 (%)**	**Q30 (%)**	**GC (%)**
C	21,954,068	21,547,376	6.46 Gb	0.03	97.39	92.62	35.70
Cd_24h	21,223,383	20,825,317	6.25 Gb	0.03	97.49	92.85	36.74
Cd_21d	20,919,291	20,566,349	6.17 Gb	0.03	97.42	92.68	35.74

C, Cd_24h and Cd_21d denote the 0 h, 24 h and 21 d libraries, respectively. Each library contains RNA from nine clams sampled across three tanks. Read pair counts correspond to paired-end 150 bp sequencing; clean bases are expressed in Gb. The assembly contained 369,091 transcripts and 129,639 unigenes, with N50 values of 1,128 and 1,132 bp, respectively. Unigene BUSCO categories were 828 complete single-copy (84.7%), 17 complete duplicated (1.7%), 113 fragmented (11.6%) and 20 missing (2.0%).

Table S2. qRT-PCR primer sequences, amplicon lengths and amplification efficiencies.

Three tank-specific biological RNA samples were separately reverse-transcribed at each time. Relative expression was normalized to the geometric mean of β-actin and GAPDH and calibrated to 0 h. Module names denote functional annotation groups.

**Gene**	**Module**	**Forward primer (5′-3′)**	**Reverse primer (5′-3′)**	**Amplicon (bp)**	**Efficiency (%)**
BAX	Apoptosis	GACATCTTCGCTGAGGGAGT	TGTCCAGCTCCATGTTGTTG	142	98.6
CASP7	Apoptosis	CTGGCATCGTGTCCTACTCA	GCAGGTCCACTTCTGTTGGA	156	101.2
MT	Detoxification	ATGGACCCTTGCAACTGCAA	GCACTTGCAGTCAGCAGTTG	128	96.4
SOD	Antioxidant	TGGAGACCTGGGAGATGTGA	CCAATGATGCCCTCAGTGAC	134	99.1
TLR	Immune	CAGTGCTCAACGTGACCTTC	ATGGCAGGCTTGAAGTTGTC	148	102.5
TNN	Contractile	GCTGAAGAGCTGGAGAAGGA	CTTCTCCTGCTCGTTGAGGT	139	97.8
TPM	Contractile	AGGAGCTGGACAAGTACTCC	TCAGCCTTCTGAGCCTTCTC	151	100.4
NKA	Ion transport	TGGTGCTGTCAACGAGTTTG	ACCAGGAAGCCGATGATGAC	145	95.7
KCN	Ion channel	CTGGTCATCTTCGGCAGCTT	GAAGGTGGTGATGGCGTTGA	137	98.9
HCN	Ion channel	ATCGTGCTGAACTGCAGGAA	TGCTGTCCAGGAACTTGTCC	132	101.7
β-actin	Reference	TGCGTGACATCAAGGAGAAG	GTGGTCTCGTGGATACCGTC	130	100.8
GAPDH	Reference	AAGGTCATCCCAGAGCTGAA	CTGGGATGATGTTCTGGGTT	126	99.3

Table S3. Expression-category counts for selected KEGG annotations in the 24 h and 21 d comparisons with 0 h.

The annotation total counts unique assembly features assigned to each category. Higher and lower categories require positive FPKM in both libraries, FPKM ≥1 in at least one and an exposed/0 h ratio ≥2 or ≤0.5, respectively. Features detected in one library alone are shown separately. These categories summarize the pooled expression profiles. Complete GO and KEGG counts are available in Supplementary Data S2.

Time	KEGG annotation	All annotated	Higher ≥2-fold	Lower ≥2-fold	Exposed only	0 h only
24 h	Glycerolipid metabolism	81	33	2	0	1
24 h	Lysosome	306	89	11	13	1
24 h	Antigen processing and presentation	59	17	4	0	2
24 h	Pancreatic secretion	122	31	8	4	4
24 h	Fat digestion and absorption	93	34	5	4	1
21 d	Glycerolipid metabolism	81	15	15	3	6
21 d	Lysosome	306	63	62	6	11
21 d	Antigen processing and presentation	59	19	17	0	4
21 d	Pancreatic secretion	122	28	12	9	4
21 d	Fat digestion and absorption	93	24	14	3	2

Table S4. Mortality in the 96 h acute Cd exposure test.

Nominal Cd concentration (mg/L)	**Mortality (%)**	Total deaths / 45
0 (Control)	0.00 ± 0.00	0
0.6	22.22 ± 7.70	10
1.1	24.44 ± 10.18	11
2.0	31.11 ± 7.70	14
3.6	46.67 ± 17.64	21
6.6	51.11 ± 13.88	23
12	86.67 ± 6.67	39

Mortality is mean ± SD across three tanks of 15 clams per concentration. Total deaths were calculated from the reported mean percentages and the initial 45 clams at each concentration. The probit estimate of the 96 h LC_{50} was 3.708 mg/L (95% confidence interval, 2.780–5.213 mg/L).

Supplementary Figures

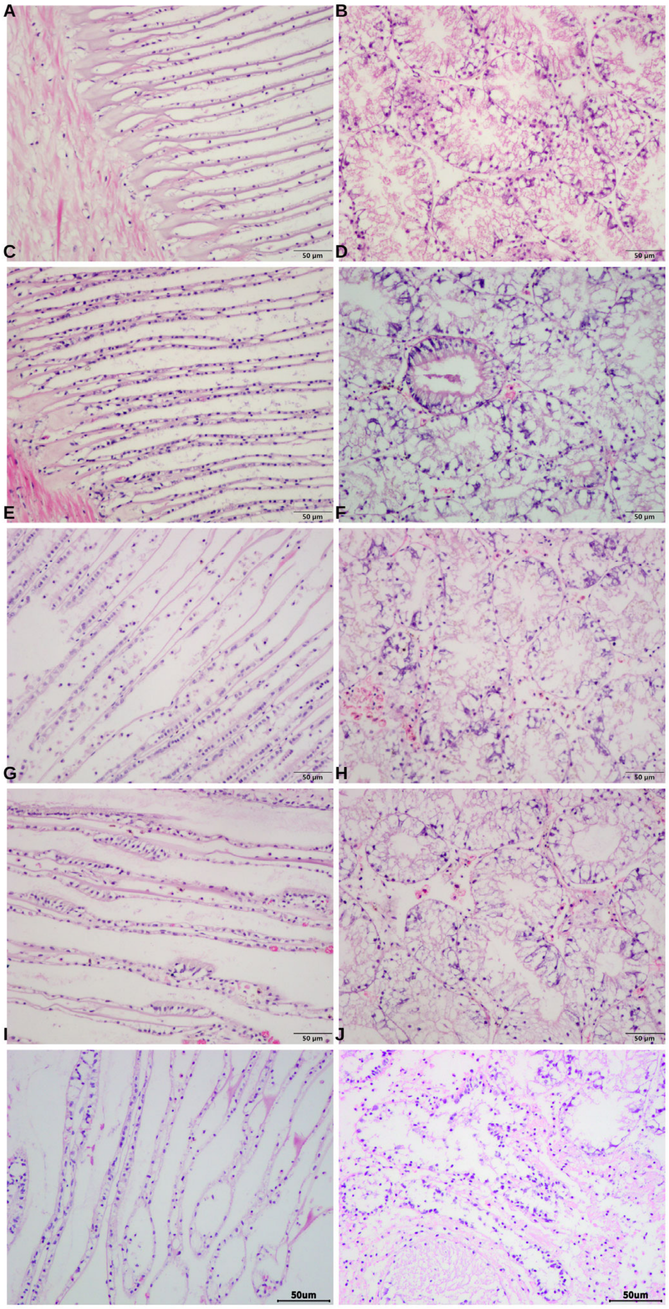

Figure S1. Hematoxylin and eosin (H&E)-stained sections of the gill and hepatopancreas from the main experiment. Left and right columns show gill and hepatopancreas; rows represent 0, 24, 96, 168 and 480 h (A–J). Nine clams were sampled per condition and time, three from each tank. Five sections were prepared per animal and tissue; one was randomly selected for imaging. Selection and assessment were blinded to treatment. Scale bars are shown in the panels.

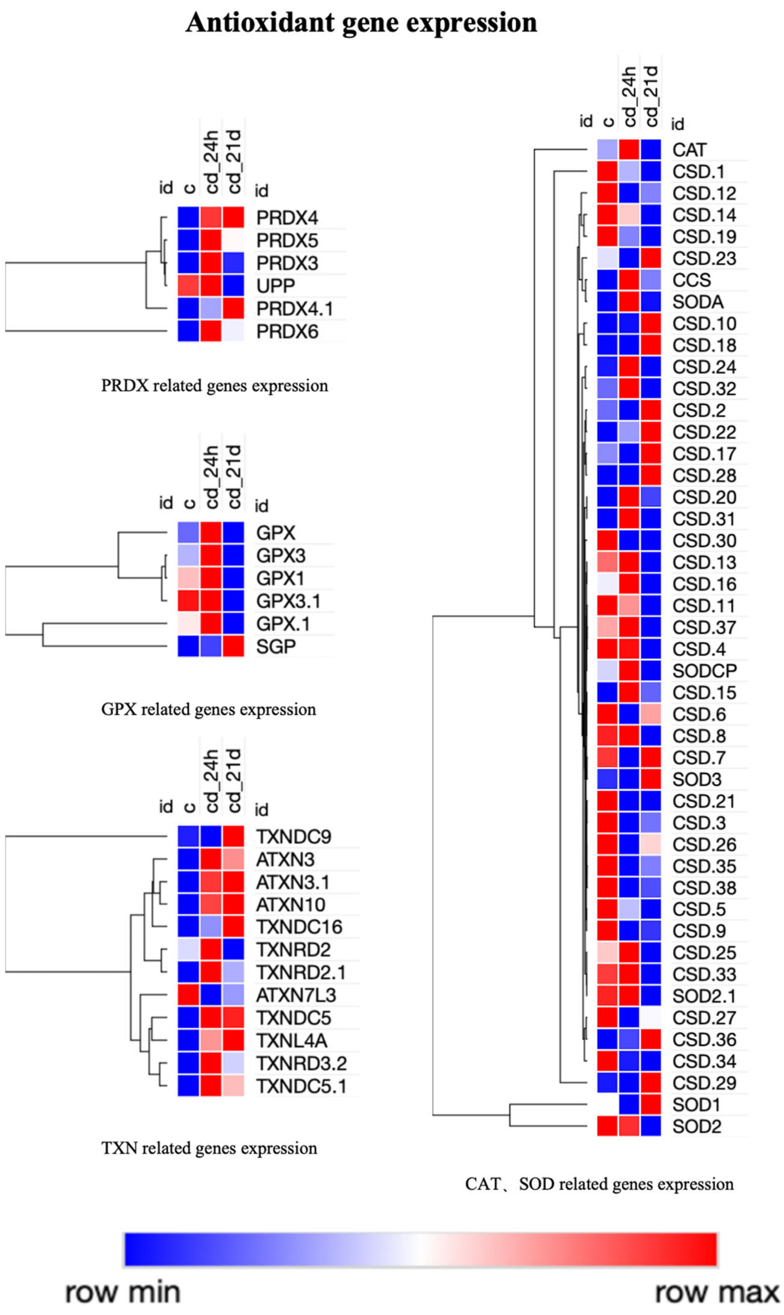

Figure S2. Hepatopancreas expression heatmaps for antioxidant-associated annotations. C, Cd_24h and Cd_21d represent the individual pooled libraries at 0 h, 24 h and 21 d. Colors show the relative expression range within each row after minimum-to-maximum scaling. Rows are grouped by functional annotation.

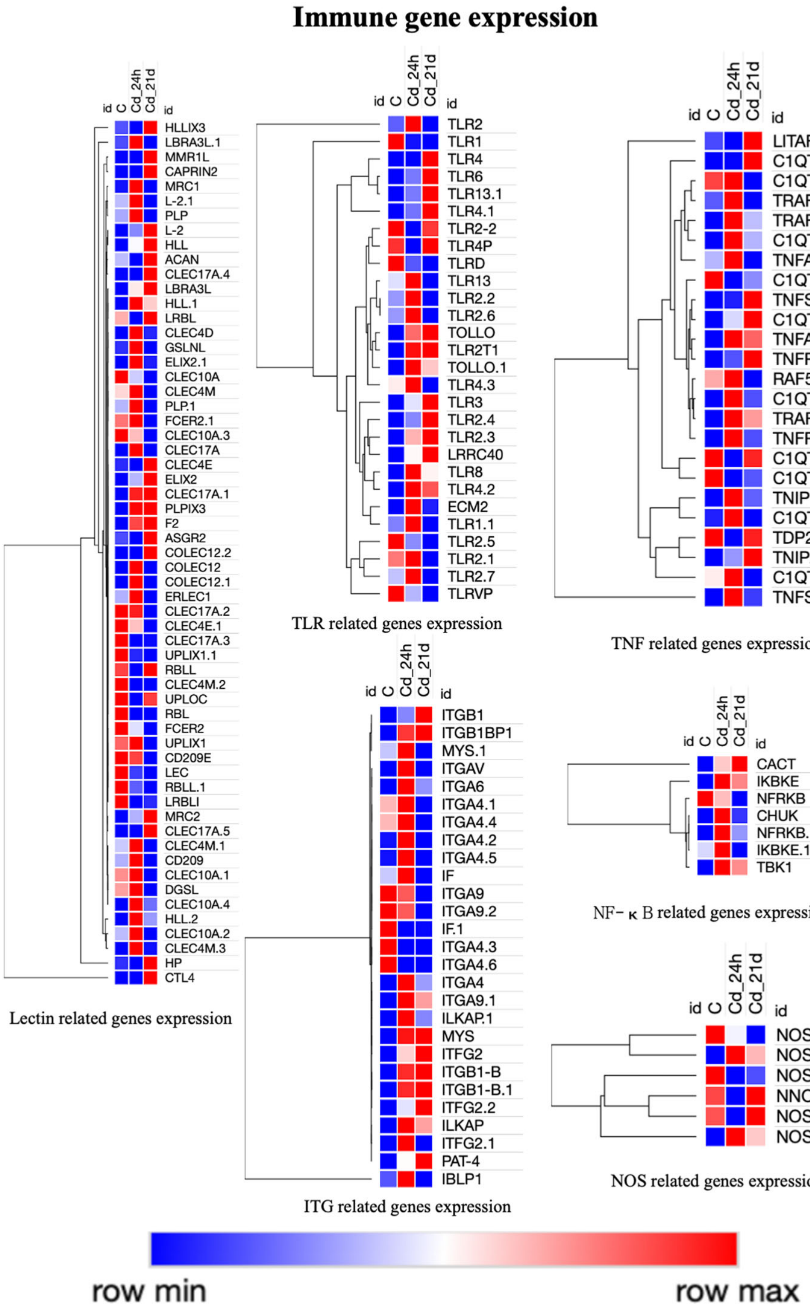

Figure S3. Hepatopancreas expression heatmaps for immune-associated annotations. Library labels and within-row scaling are as defined in Figure S2.

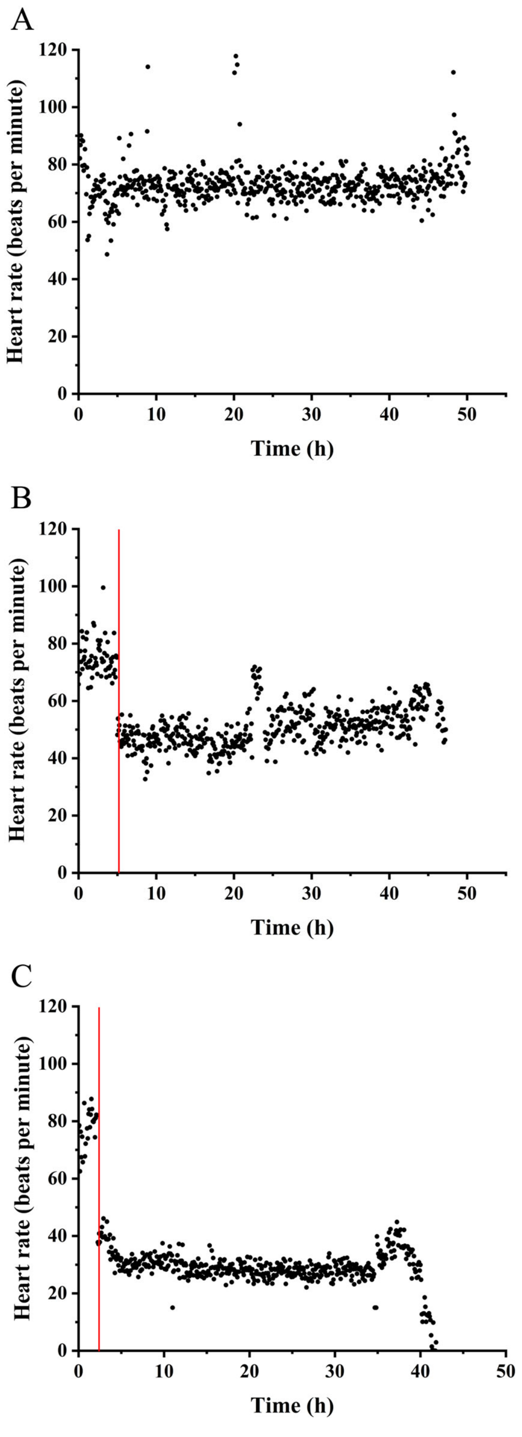

Figure S4. Representative individual heart-rate recordings during the 48 h assay: control (A), 5 mg/L Cd (B) and 20 mg/L Cd (C). Sixteen clams per concentration were instrumented in two batches of eight. Each panel shows one individual trace. Measurements used a 30 L water volume, with no feeding or water renewal during recording.

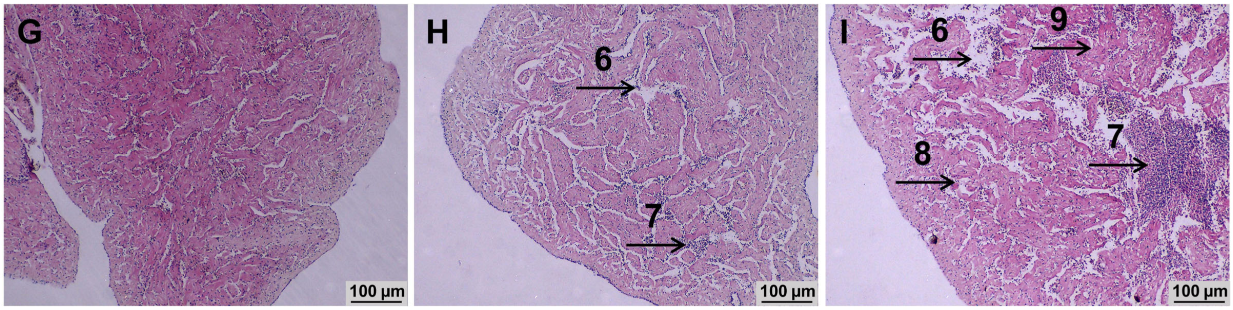

Figure S5. Representative hematoxylin and eosin (H&E)-stained heart sections at the end of the 48 h cardiac assay. Original labels G, H and I denote control, 5 mg/L Cd and 20 mg/L Cd, respectively. Six living animals per concentration supplied heart tissue. Arrows indicate changes in fiber organization, interstitial spaces and cellular infiltration. Scale bars are shown in the panels.
